# hMMS2 serves a redundant role in human PCNA polyubiquitination

**DOI:** 10.1186/1471-2199-9-24

**Published:** 2008-02-19

**Authors:** Jan Brun, Roland Chiu, Katherine Lockhart, Wei Xiao, Bradly G Wouters, Douglas A Gray

**Affiliations:** 1 Centre for Cancer Therapeutics, Ottawa Health Research Institute, Ottawa, ON, K1H 8L6, Canada; 2 Department of Biochemistry, Microbiology, and Immunology, University of Ottawa, Ottawa, Canada; 3 Department of Radiation Oncology (Maastro Lab), GROW Research Institute, University of Maastricht, Maastricht, The Netherlands; 4 Department of Microbiology and Immunology, University of Saskatchewan, Saskatoon, Canada

## Abstract

**Background:**

In yeast, DNA damage leads to the mono and polyubiquitination of the sliding clamp PCNA. Monoubiquitination of PCNA is controlled by RAD18 (E3 ligase) and RAD6 (E2 conjugating enzyme), while the extension of the monoubiquitinated PCNA into a polyubiquitinated substrate is governed by RAD5, and the heterodimer of UBC13/MMS2. Each modification directs a different branch of the DNA damage tolerance pathway (DDT). While PCNA monoubiquitination leads to error-prone bypass via TLS, biochemical studies have identified MMS2 along with its heteromeric partner UBC13 to govern the error-free repair of DNA lesions by catalyzing the formation of lysine 63-linked polyubiquitin chains (K63-polyUb). Recently, it was shown that PCNA polyubiquitination is conserved in human cells and that this modification is dependent on RAD18, UBC13 and SHPRH. However, the role of hMMS2 in this process was not specifically addressed.

**Results:**

In this report we show that mammalian cells in which MMS2 was reduced by siRNA-mediated knockdown maintains PCNA polyubiquitination while a knockdown of RAD18 or UBC13 abrogates PCNA ubiquitination. Moreover, the additional knockdown of a UEV1A (MMS2 homolog) does not deplete PCNA polyubiquitination. Finally, mouse embryonic stem cells null for MMS2 with or without the additional depletion of mUEV1A continue to polyubiquitinated PCNA with normal kinetics.

**Conclusion:**

Our results point to a high level of redundancy in the DDT pathway and suggest the existence of another hMMS2 variant (hMMSv) or complex that can compensate for its loss.

## Background

Protecting the integrity of the DNA genome is important for the long-term survival of higher eukaryotes [1]. Given the importance of genomic integrity, it is not surprising that an elaborate system of cell cycle checkpoints and DNA repair systems has evolved in higher vertebrates. However, the failure to remove DNA lesions in a timely and efficient manner often forces a cell to bypass the damage in order to avoid replication stalling. This is accomplished through DNA damage tolerance (DDT), an important component of the DNA damage response. DDT allows replication machinery stalled at sites of DNA damage to continue with DNA synthesis by allowing bypass of such sites in either an error-prone or error-free manner [2]. The control of such a pathway is dependent on the modification status of the sliding clamp PCNA.

In the absence of DNA damage, yeast PCNA is typically sumoylated on K164 which promotes normal S phase progression by preventing unwanted recombination, while replication stress results in PCNA ubiquitination on the same lysine residue [3,4]. There is a central role for Rad6 (ubiquitin conjugating E2 enzyme) and Rad18 ubiquitin ligase (E3 ligase) in PCNA ubiquitination. During genotoxic stress, Rad6 is recruited by Rad18 to sites of DNA damage where it monoubiquitinates PCNA [4]. Monoubiquitinated PCNA facilitates the recruitment of error-prone translesion polymerases including polymerase η, and Rev1 [5,6]. While undesirable, the mutations induced by this error-prone mechanism may be less deleterious than catastrophic blockage of replication forks. However, there is also an alternate complex (Ubc13/Mms2/Rad5) that forms and mediates error-free bypass by extending the monoubiquitinated PCNA via K63-polyubiquitin chains [4]. The outcome of this polyubiquitination has yet to be fully elucidated; it clearly does not involve the proteasome but more likely involves fork reversal, recombination with an undamaged sister chromatid either at or behind the replication fork [4,7].

A majority of the genes of the DDT pathway have been conserved from yeast to human including RAD18, RAD6, MMS2, UBC13, PCNA and most of the translesion polymerases [8,9]. As such, Kannouche et al., and Watanabe et al., have shown that the error-prone arm of DDT involving PCNA monoubiquitination and concordant polymerase switching is fully conserved in mammalian cells [10,11]. Recently, we reported that PCNA is polyubiquitinated in human cells after DNA damage [12,13]. Accordingly, PCNA polyUb was shown to be dependent on RAD18 (suggesting that monoubiquitination of PCNA is required), and also on UBC13 (the ubiquitin conjugase previously shown to be involved in K63-polyubiquitin chain formation) [12]. Recently, Motegi et al and Unk et al. have confirmed the existence of polyubiquitinated PCNA and have suggested that SHPRH (human ortholog of yeast RAD5) is the E3 ligase involved this process [14,15]. However, it has yet to be determined whether hMMS2 (heteromeric partner of hUBC13) participates significantly in PCNA polyubiquitination in human cells.

MMS2 is a ubiquitin conjugating enzyme variant (UEV) protein that resembles ubiquitin conjugating enzymes (E2s) but lacks a conserved active cysteine site [16]. Based on this original observation, it was hypothesized that UEV proteins behaved as negative regulators of ubiquitination. However, biochemical evidence showed that MMS2 forms a heteromeric complex with UBC13 (an E2 conjugating enzyme with active catalytic cysteine site) to catalyze the formation of K63-polyubiquitin chains [17,18]. In addition, loss of MMS2 in yeast resulted in overt sensitivity to a variety of genotoxic agents as well as increased spontaneous and UV induced mutagenesis confirming its role in the error-free damage avoidance arm of DDT [18,19]. Similar to the findings in yeast, the expression of anti-sense MMS2 in human cells resulted in increased UV induced mutagenesis [20]. These data provide strong evidence of the importance of MMS2 in the assembly of polyubiquitin chains linked through K63 in the error free damage avoidance arm of the DDT pathway in both yeast and humans.

Since MMS2 serves such an important role in error free DNA repair in both humans and yeast and has been shown to be indispensable in yeast PCNA polyubiquitination, we predicted that it would be equally important in PCNA ubiquitination in mammalian cells. Therefore, we examined the state of PCNA ubiquitination in mouse MMS2 knock-out embryonic stem cells and human cells in which hMMS2 had been depleted by siRNA. Unlike budding yeast which has only one UEV gene (Mms2), human cells contain at least 4 UEV loci. In this paper, we address the role of hMMS2 and hUEV1a, the most likely functional orthologues of yeast MMS2, in promoting PCNA polyubiquitination. We report that PCNA polyubiquitination proceeds with normal kinetics in the presence or absence of MMS2 whereas UBC13 or RAD18 knockdown abrogates PCNA polyubiquitination. Additionally, depletion of UEV1a does not disrupt PCNA polyubiquitination after DNA damage. This suggests that there is a high degree of redundancy built into mammalian systems and the potential for the existence of a MMS2 variant that can compensate for the loss of hMMS2.

## Methods

### Cell culture and treatments

The mouse embryonic MMS^+/+ ^or MMS^-/- ^stem cells (kindly provided by Dr. Wei Xiao, University of Saskatchewan) were cultured in DMEM supplemented with 15% FBS, 1X pen/strep, LIF (kindly provided by Dr. M. McBurney, Ottawa Health Research Institute) and beta-mercaptoethanol (EMD Chemicals, Omnipur, Gibbstown New Jersey, United States). The HEK 293T and Hela cell lines were cultured in DMEM (Gibco, Invitrogen, Carlsbad, California, United States) supplemented with 10% FBS (Gibco, Invitrogen, Carlsbad, California, United States). 293T and Hela cells were transfected with siGENOME SMARTpool reagent specific for human RAD18, UBC13, MMS2 and/or hUEV1a (Dharmacon Research, Lafayette, Colorodo, United States) using oligofectamine (Invitrogen Carlsbad, California, United States). The transfections were performed 72 hours prior to harvesting the cells to achieve optimal long-term knockdown as determined by immunoblotting. UV irradiation (30 J/m^2^) was performed on exponentially growing cells using a UVC germicidal lamp at a fluence rate of 1 J/m^2^/s.

### Western blotting

RNA interference and the preparation of proteins lysates were performed as described previously [12]. Cells transfected with siGENOME SMARTpool reagent specific for human UBC13, human RAD18 human MMS2 or human UEV1A (Dharmacon Research, Lafayette, Colorodo, United States) were UV irradiated and lysed 6 h post-treatment. Samples were sonicated, soluble fractions were recovered, and proteins were quantified. Proteins were resolved on either a one phase or two phase SDS-polyacrylamide gel (10% or 10% and 15%) and electroblotted onto a Hybond C nitrocellulose membrane (Amersham Pharmacia Biotech, Piscataway, New Jersey, United States). The following antibodies were used: mouse monoclonal (mAb2h11) that recognizes both hMMS2 and UEV1a [21], mouse monoclonal UBC13 (mAb 4E11) [21], mouse monoclonal PCNA PC10 (Millipore, Chemicon, Temecula California), and mouse monoclonal actin (Sigma, St. Louis Missouri, United States). Proteins were visualized using SuperSignal West Pico Chemiluminescent Substrate (Pierce Biotechnology Rockford, Illinois, United States).

### Immunoprecipitations

PCNA immunoprecipitations were carried out as described previously [12,13]. Brielfy cells were UV-irradiated with 30 J/m2 as described above and either left untreated or transfected with SiGenome Smartpool reagent specific for human RAD18, human MMS2 and/or human UEV1a (Dharmacon). An anti-PCNA antibody was incubated overnight with 500 μg of protein lysate. The following day, lysates were incubated with 100 μL of Gamma-Bound Sepharose Beads (Amersham Pharmacia Biotech). After 48 h beads were washed extensively in lysis buffer, and proteins were eluted by boiling in Laemmli's SDS sample buffer. Immunoblotting was performed as described above except that the PVDF membranes (Millipore) were used and autoclaved for 20 min in ddH2O after protein transfer, and proteins were visualized using chemiluminescent substrate (Amersham Pharmacia).

### RNA Extractions

E14K mouse embryonic stem (ES) cells (wild type and *mMms2*-/-) were grown on 100 mm tissue culture dishes coated with 1% gelatin. Cells were cultured at 37°C in a 5% CO_2 _atmosphere, in cell medium containing high glucose D-MEM (Sigma) supplemented with 15% fetal bovine serum, 100 U/ml penicillin, 100 μg/ml streptomycin, 0.1 mM MEM non-essential amino acids (all from Invitrogen), 0.05 mM 2-mercaptoethanol (Sigma), and 1,000 U/ml LIF (ESGRO; Chemicon). Prior to total RNA extraction, cells were trypsinized and collected. The cells were lysed in Trizol (Invitrogen) and total RNA was isolated using the manufacturer's suggested protocol. Prior to cDNA synthesis, the RNA samples were DNase I treated using Ambion's DNA-*free *kit as per the manufacturer's suggested protocol. Total RNA (2 μg) was reversed transcribed using oligo(dT)_20 _and Invitrogen's Thermoscript RT-PCR system as per the manufacturer's protocol. The PCR step was subsequently carried out using 2 μl of the RT reaction, 1.25 μM of each specific primer, and 2.5 U of Platinum Taq polymerase (Invitrogen) in a 40 μl reaction. Both mMms2 and β-actin control PCR reactions were completed under the following conditions: an initial denaturation at 94°C for 2 minutes, a subsequent denaturation step at 94°C for 30 seconds, an annealing temperature of 50°C for 30 seconds, and an extension step at 72°C for 20 seconds. This was repeated for a total of 32 cycles and 26 cycles for mMms2 PCR and β-actin control PCR respectively, with a final extension of 72°C for 10 minutes. The sequences of the Mms2 forward and reverse primers are, respectively, 5'-GGCAGTCTCCACAGGAGTTAAA-3' and 5'-ACTGGAATTATTGATCCCATTCA-3' to yield a 283 bp product. The sequences of the β-actin control PCR forward and reverse primers are 5'-AGGTCATCACTATTGGCAACGA-3' and 5'-AGTACTTGCGCTCAGGAGGA-3' respectively, to yield a 276 bp product.

## Results

### Efficient Knockdown of MMS2 and UEV1a

**Figure 1:**
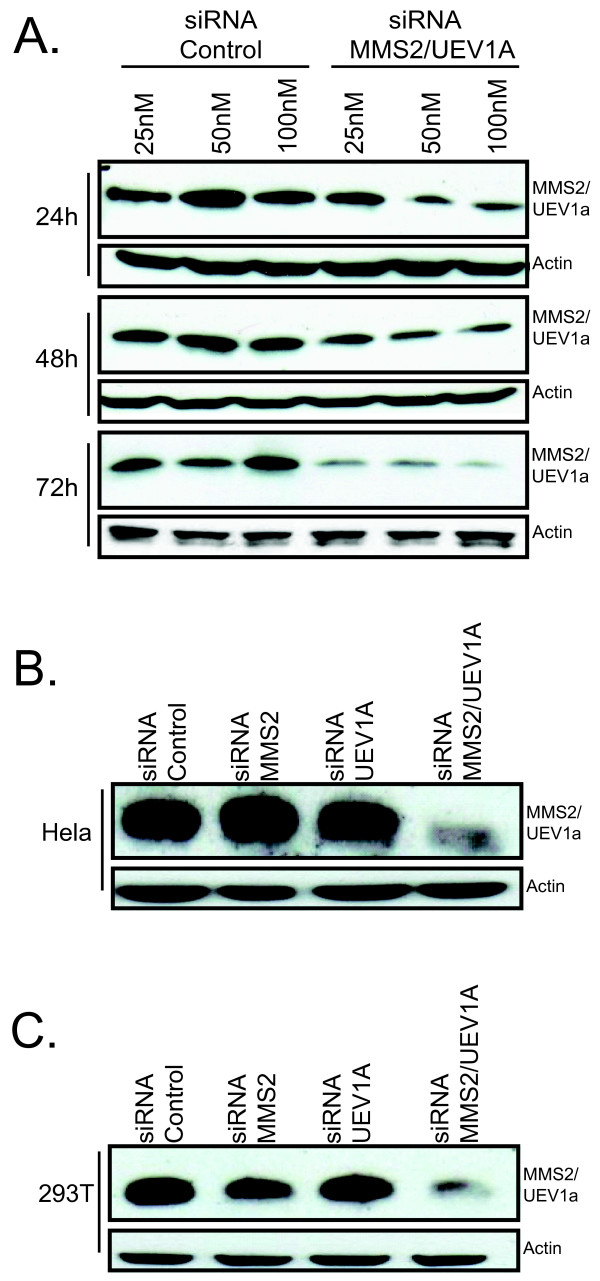
**siRNA targeting MMS2 and UEV1a results in an efficient knockdown**. **(A) **293T cells were transfected with 100 nM of either control siRNA, siRNA MMS2 and siRNA UEV1A followed by immunoblotting. Cells were lysed at the indicated time points and subject to immunoblotting. **(B) **Hela cells were treated as above and lysed 72 hours post-transfection. **(C) **293T cells were treated as in (B).

In yeast, monoubiquitination requires the ubiquitin E2/E3 complex RAD6/RAD18 and further polyubiqutination is dependent on the RAD5/UBC13/MMS2 complex. In mammalian cells, RAD18 has been implicated in monoubiquitination, and SHPRH and UBC13 in polyubiquitination of PCNA [12,14,15]. To ascertain whether mammalian MMS2 is also required for polyubiquitination we targeted MMS2 in HEK 293T and Hela cells using siRNA (Figure 1). The Mms2 antibody used in our studies recognizes both Mms2 and the other mammalian homolog Uev1a [21]. Therefore to confirm effective knockdown with this antibody we performed a double knockdown with siRNA targeting both hMMS2 and hUEV1a. The abundance of MMS2 and UEV1a was found to be effectively reduced 72 hours post- transfection in both 293T and Hela cells suggesting that both individual knockdowns were effective (Figure 1A–C).

### PCNA is polyubiquitnated in the absence of MMS2 and/or UEV1a

**Figure 2:**
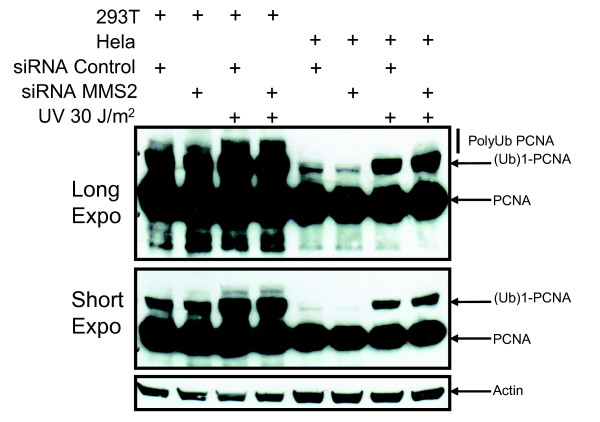
**MMS2 Knockdown does not disrupt PCNA polyubiquitination**. 293T and Hela cells were transfected with 100 nM of either control siRNA or siRNA specific for MMS2. 72 hours later cells were UV irradiated with either 0 or 30 J/m^2 ^and lysed 6 hours post-treatment followed by immunoblotting with an anti-PCNA antibody.

**Figure 3:**
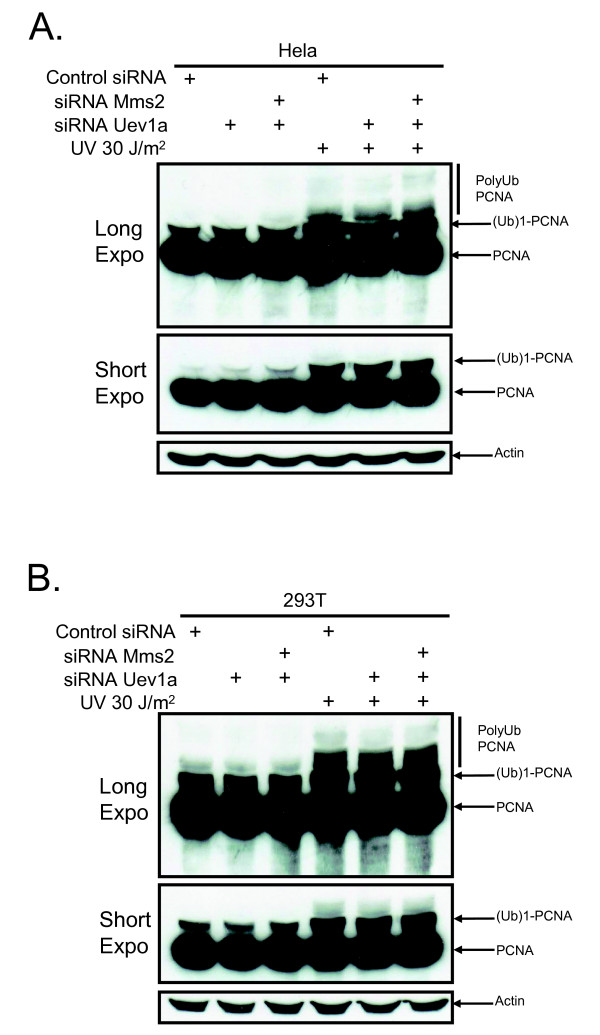
**A knockdown of MMS2 and UEV1a does not abrogate PCNA polyubiquitination**. **(A) **Hela cells were treated as in Figure 2 except they were additionally transfected with siRNA UEV1A. **(B) **293T cells were treated as in Figure 3A.

**Figure 4:**
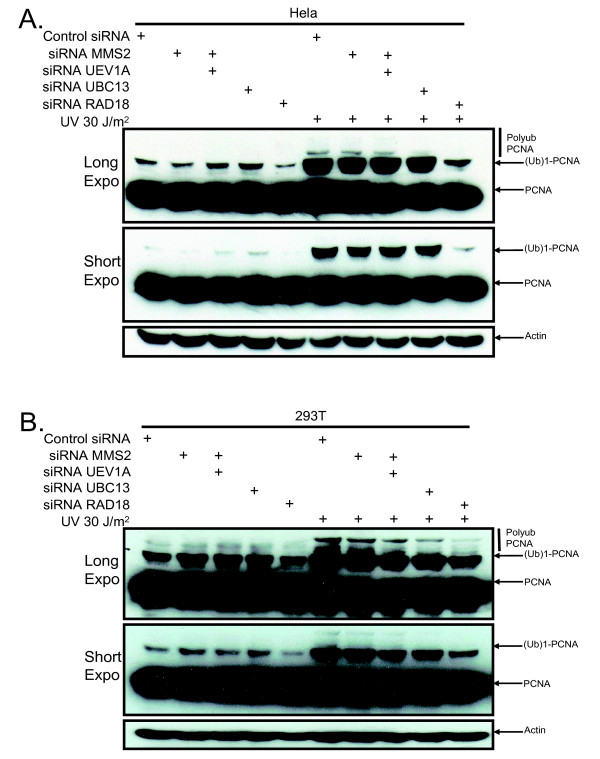
**RAD18 or UBC13 knockdown disrupts PCNA polyubiquitination**. **(A) **Hela cells were treated the same as in Figure 3 except that cells were also transfected with siRNA targeting UBC13 and RAD18. **(B) **293T cells were treated as in Figure 4B.

Cells treated with siRNA against MMS2, UEV1a, or both were exposed to UV light to induce DNA damage and then assessed for ubiquitination of PCNA. As expected, the band corresponding to monoubiquitinated PCNA was unaffected by MMS2 knockdown after UV irradiation (Figure 2). Unexpectedly, knockdown of MMS2 alone did not affect the higher molecular weight bands corresponding to polyubiquitinated PNCA (Figure 2). Since MMS2 belongs to a family of UEVs that have been conserved throughout evolution in higher eukaryotes [16,22-24] we speculated that one of these UEVs could compensate for its loss. Human UEV1A is an obvious candidate since it can bind to UBC13 and has greater than 90% amino acid sequence identity with hMMS2 [25]. However, our results demonstrated little or no reduction in polyubiquitinated PCNA after knockdown of UEV1a alone or in combination with MMS2 (Figure 3A and 3B; Additional file 1(A) and 1(B)). This is in contrast to knockdown of UBC13 (Additional file 2(A) and 2(B)) or RAD18 (Figure 4A and 4B) which effectively blocked PCNA polyubiquitination (Figure 4A and 4B; Additional file 2(C) and 2(D)). These findings suggest that MMS2 likely serves a redundant role in PCNA polyubiquitination.

### Abrogation of MMS2 and/or UEV1A does not alter ubiquitin laddering

**Figure 5:**
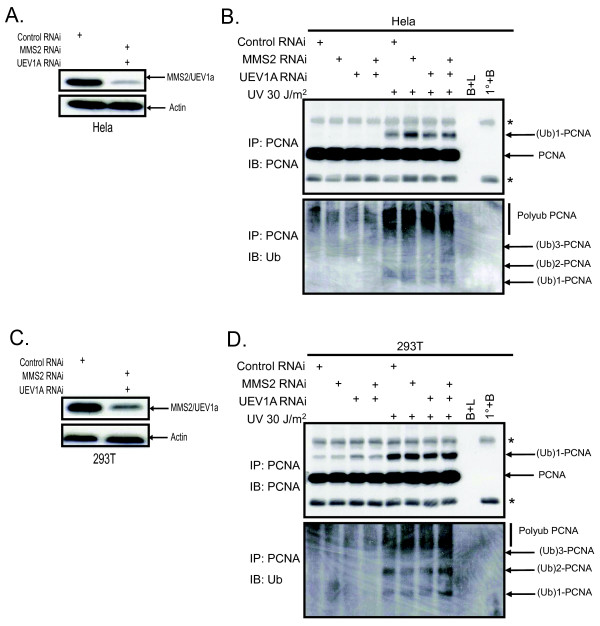
**PCNA ubiquitin laddering is not altered by the knockdown of MMS2 and UEV1a**. **(A) **Hela and **(C) **293T cells were treated as in Figure 1 followed by immunoblotting for MMS2 and UEV1a **(B) **Hela and **(D) **293T cells were transfected as described in figure 1. Seventy-two hours post-transfection, cells were irradiated with 30 J/m^2 ^of UV and lysed 6 h later in boiling SDS, diluted in lysis buffer, and subjected to immunoprecipitation with a PCNA antibody and immunoblotted for PCNA (upper panel) and Ub (lower panel). The controls in the immunoprecipitations were "B+L", in which lysates were incubated with beads but no PCNA antibody, and "1°+B" in which PCNA antibody was incubated with beads alone. Asterisks denote immunoglobulin heavy and light chains as detected in the immunoprecipitations.

**Figure 6:**
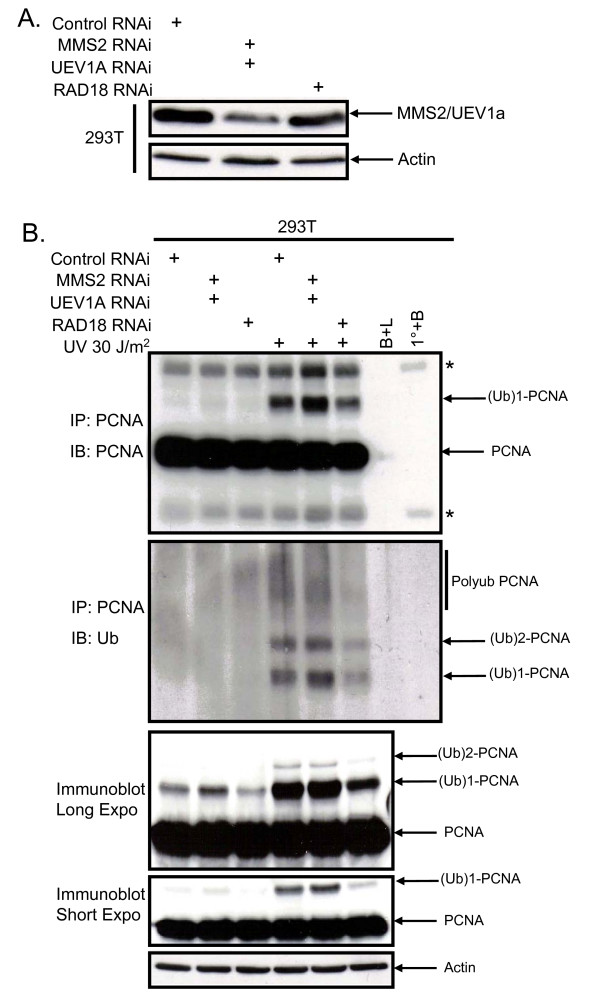
**PCNA ubiquitination decreases after RAD18 knockdown**. **A) **293T cells were transfected with 100 nM of either control siRNA, MMS2 and UEV1A siRNA or RAD18 siRNA followed by immunoblotting. **B) **Seventy-two hours post-transfection, cells were subjected to immunoprecipitation with a PCNA antibody and immunoblotted for PCNA (upper panel) and Ub (lower panel). A PCNA immunoblot with darker and lighter exposure performed on protein lysates from the same samples used in the immunoprecipitations is also shown. Asterisks denote immunoglobulin heavy and light chains as detected in the immunoprecipitations.

Thus far our immunoblotting data supports the notion that MMS2 serves a redundant role in PCNA ubiquitination. However, to confirm that these bands are indeed ubiquitinated we knocked down MMS2 and UEV1A using siRNA and performed a PCNA immunoprecipitation followed by immunoblotting for ubiquitin. Despite the efficient reduction in MMS2 and UEV1A (Figure 5A and 5C), several bands corresponding to mono, di, tri and tetra ubiquitinated species were observed in the double knockdown lane which was indistinguishable from either of the single knockdown or the control lane (Figure 5B and 5D and Additional files 3(A) and 3(B)). Importantly, this laddering pattern was similar to the ones observed from previous studies after treatment with MMS, BPDE or UV light [12,13,15,26]. Moreover, a knockdown of RAD18 in 293T cells demonstrates a decreased pattern of polyubiquitinated PCNA as compared to the double knockdown of MMS2 and UEV1A or cells transfected with control siRNAs (Figure 6A and 6B). Finally, PCNA immunoblotting data (Figure 6B bottom 3 panels) using the same lysates were consistent with the data obtained from IP's in Figure 5, and figure 6B (upper 2 panels).

### MMS2^-/- ^knockout stem cells maintain PCNA polyubiquitination

**Figure 7:**
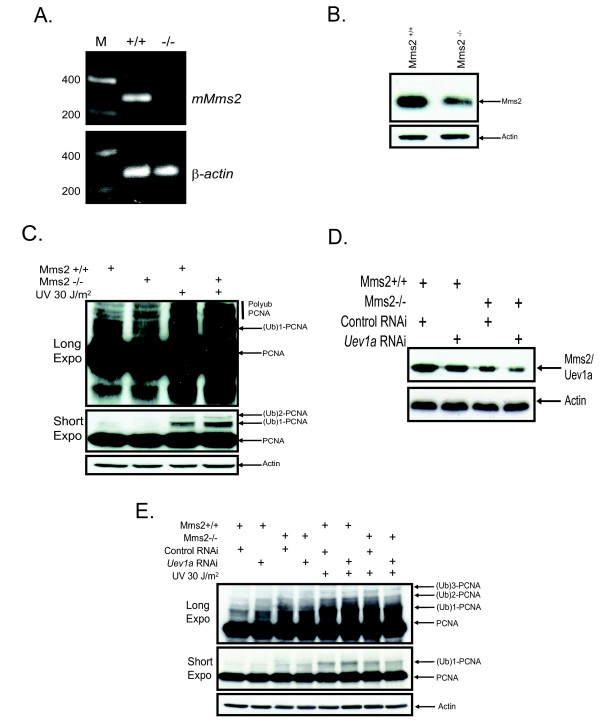
**MMS2 null mouse emybronic stem show no overt disruption of PCNA polyubiquitination after UV irradiation**. **(A) **RT-PCR for MMS2 was performed as described in material and methods **(B) **Wild type and MMS2 null embryonic stem cells were lysed and subjected to immunoblotting with anti-Mms2/Uev1a antibody. **(C) **Confluent plates of wild type and MMS2 null embryonic stem cells were split 1:4 on Day 1. On Day 2 media was replaced and followed by UV irradiation with either 0 or 30 J/m^2 ^on Day 3. Cells were harvested 6 hours post-treatment and subjected to immunoblotting with an anti-PCNA antibody. **(D) **Wild type and MMS2 null embryonic stem cells were transfected with *Uev1a *and lysed 72 hours later followed by immunoblotting with anti-Mms2/Uev1a antibody. **(E) **MMS2-/- cells were transfected with siRNA targeting mouse UEV1a. 72 hours post transfection cells were UV irradiated, lysed and subjected to immunoblotting using an anti-PCNA antibody.

In order to confirm the results of our study in human cells and rule out the possibility of off target effects of siRNA, we obtained mouse embryonic stem cells in which the *Mms2 *gene had been inactivated by gene targeting. RT-PCR reactions confirmed the absence of the MMS2 mRNA (Figure 7A). Interestingly, loss of *Mms2 *results in an approximate 50% reduction in the intensity of the MMS2/UEV1a band detected (Figure 7B). The latter is expected since the *Uev1a *levels remain the same and the antibody recognizes both *Mms2 *and *Uev1a*. Monoubiquitination of PCNA after UV treatment occurred normally and with identical kinetics in the *Mms2*-/- cells compared to wild type. Consistent with our siRNA results in Figures 2, 3, 4, we did not observe a reduction in PCNA polyubiquitination in the *MMS2 *knock-out cells (Figure 7C). Finally, when we targeted *Uev1a *in the *Mms2-/- *cells PCNA ubiquitination was not abrogated (Figure 7D and 7E). In fact it occurred with normal kinetics as those seen in the wildtype ES cells and as observed in our human cell lines.

## Discussion

UEVs such as MMS2 and UEV1a constitute a highly conserved family of distinct E2 conjugating enzymes devoid of a catalytic cysteine [16,20,24]. Functionally they act in complexes with a heteromeric partner such as UBC13. The MMS2/UBC13 or UEV1a/UBC13 heterodimer catalyzes the formation of non-canonical K63-polyubiquitin chains that are involved in DDT and activation of NF-κB respectively [21]. Since MMS2 has been implicated in the error-free lesion bypass in yeast and humans and in light of the recent data demonstrating PCNA polyubiquitination after DNA damage in human cells [12,15], we sought to determine whether hMMS2 plays an important role in polyubiquitinating PCNA in mammalian cells.

In the present study we demonstrate that cells partially or totally devoid of MMS2 (through siRNA or gene targeting) and cells experiencing knockdown of both MMS2 and UEV1a do not exhibit a significant reduction in PCNA polyubiquitination after UV irradiation. These results are in sharp contrast to those obtained from the knockdown of UBC13 or RAD18. In isolation, the MMS2 findings might beg the question of whether K63-polyubiquitination is important in human DNA repair. There are, however, convincing studies in the recent literature in support of the importance of hRAD18, hUBC13 and SHPRH (the human RAD5 homolog) in K63-polyubiquitination of PCNA and its role in maintaining genomic stability [12,14,15]. The most parsimonious explanation of our results would therefore invoke a built in redundancy of the MMS2 component in the higher eukaryotes.

Several lines of evidence support our contention that redundancy is the best explanation. First, although hMMS2 was shown to be involved in damage induced mutagenesis, no overt sensitivity to UV light or increased spontaneous mutagenesis was observed in human fibroblasts as it had been observed in the yeast system [20]. Second, Simpson et al., showed that disruption of MMS2 in a chicken cell line, DT 40, did not result in increased sensitivity to DNA damaging agents nor did it promote sister chromatid exchange [27]. Third, in a previous publication we demonstrated that knockdown of UBC13 or RAD18 did not entirely abrogate PCNA ubiquitination [12]. Although this could be due to incomplete knockdown, recent evidence by Simpson et al. have demonstrated that RAD18 independent PCNA ubiquitination occurs in RAD18 null DT40 cells suggesting the presence of compensatory PCNA ubiquitinating enzyme in higher vertebrates [28]. We believe this to be the case in human cells as well. Finally, we show that PCNA is ubiquitinated in the presence or absence of hMMS2. Overall, these data highlight the inherent complexity of the DDT pathway and in particular point to a greater degree of built in redundancy in vertebrates.

Given the importance of MMS2 in yeast and human cells and our current finding we asked whether another UEV gene could complement the loss of hMMS2. To our knowledge yeast contain only a single UEV locus (MMS2) while humans contain at least 4 UEV loci including UBE2V1 (also known as CROC1A, UEV1A, UEV1, CIR1), UBE2V2 (also known as hMMS2 UEV2, DD-VIT, EDAF-1, and EDPF-1), TSG101 and UEV3[25,29,30]. UBE2V1 has alternatively spliced isoforms including UEV1a (CROC1A), KUA-UEV, and Croc1B which vary in their 5' sequence [24,31,32]. Such UEVs may potentially complement hMMS2. However, based on structural studies TSG101 is an unlikely candidate to complement hMMS2 as it does not bind with UBC13 and to date has not been shown to catalyze K63 polyubiquitination [24]. UEV3 can also be eliminated as its sequence is very similar to TSG101 and it likely does not associate with UBC13 [29,31]. In addition, Thompson et al. show that KUA-UEV is strictly localized to the cytoplasm [31], excluding the protein from the site of DNA repair and thereby excluding it as a functional substitute for MMS2. Furthermore, a yeast two hybrid study eliminates CROC1b as a candidate since it failed to show interaction with UBC13 [21]. hUEV1a would appear to be a strong candidate to complement MMS2 because it can bind to UBC13, localize to the nucleus, and complement the Mms2 yeast mutant. It also has greater than 90% amino acid sequence identity with hMMS2 [25,31,32]. Therefore, we used siRNA to target UEV1a along with hMMS2. However, the combined knockdown of UEV1a and hMMS2 did not abrogate PCNA polyubiquitination in human cells. This result is consistent with two distinct cellular roles of hMMS2 (in DNA repair) and UEV1a (in NF-κB activation) as previously shown by Anderson et al. and argues against a joint or collaborative role in DDT [21].

Previous studies have clearly shown that the expression of anti-sense MMS2 in human cells results in increased UV induced mutagenesis. Our contention that MMS2 is dispensible for PCNA ubiquitination leads to some obvious questions concerning the role of MMS2 in DNA damage tolerance in human cells. However, it is possible that the function of MMS2 in human cells with respect to DNA damage tolerance is one that is independent of PCNA ubiquitination. An intriguing possibility is that it plays a direct role in homologous recombination itself. In fact this has recently been suggested in the case for its heterodimeric partner UBC13 and its E3 ligase partner RAD18 [33,34]. Both proteins seem to be playing a direct role in homologous recombination itself. While RAD18 seems to facilitate HR by suppressing NHEJ, cells deficient in UBC13 are unable to recruit the key HR proteins RPA, BRCA1, and Rad51 to sites of DNA damage. This occurs due to a failure in a very early stage of HR-mediated repair of DNA double strand breaks (DSBs) where UBC13 is required to process DSBs into a competent HR substrate. This may help explain why the PCNA-K164R mutant DT40 cells are different in certain aspects from the ones of cells deficient in RAD18 or UBC13, which implies that some aspects of DNA damage tolerance are independent of PCNA modification. Moreover, it suggests that PCNA ubiquitination might be involved in additional processes such as growth; a contention supported by the smaller size and the sub-mendelian frequency at which homologous PCNA K164R mutant mice are born [35]. Finally as shown by Motegi et al. and Unk et al., the function of MMS2 in PCNA ubiquitination might be linked to the one of SHPRH, a recently discovered functional orthologue of yeast RAD5 [14,15]. Certainly in yeast the ability of MMS2 and UBC13 to ubiquitinate PCNA largely depends on RAD5. Therefore, this distinct possibility cannot be excluded from occurring in human cells with MMS2 and SHPRH.

It is possible that complexes other than the heteromeric couple of UBC13/UEV may be able to complement the loss of hMMS2 (an example of such a complex is Np14/UFD1 which is also able to catalyze the formation of K63-polyubiquitin chains [36]) but there exists the intriguing possibility that a previously unreported MMS2 variant (herein designated hMMS2v) functionally complements MMS2 in the assembly of polyubiquitin chains on the PCNA of cells sustaining DNA damage. We have preliminary data indicating that such a variant is present at low levels in HEK 293 cells (Brun et al., unpublished). If response to DNA damage is compartmentalized then even low levels of the hMMS2 variant could potentially compensate for the loss of MMS2. However, whether this variant form is resistant to the siRNA used in this paper or if it is absent in the mouse embryonic knock cells remains to be determined. Further investigations will be required to determine the ubiquity of hMMS2v, its role in DNA repair, whether it contains UBC13 binding domain, and whether siRNA targeting of both hMMS2 and hMMS2v will fully abrogate PCNA ubiquitination.

## Conclusion

In a previous study we demonstrated that K63-linked polyubiquitination guards against environmental mutagenesis and specifically that the target of this ubiquitination PCNA is dependant on RAD18 and UBC13 [12]. It would be reasonable to assume that as the heteromeric partner of UBC13, MMS2 would be directly involved in PCNA polyubiquitination, but the data reported herein are not consistent with this prediction. On the contrary MMS2 and UEV1A seem to serve redundant roles in PCNA polyubiquitination suggesting the existence of an alternative MMS2 variant or novel E2 that can complement the loss of MMS2. Although our work does not identify the redundant protein, we believe the redundancy is in itself an unexpected and important finding that will spur further investigation.

## Abbreviations

DDT, DNA damage tolerance; TLS, translesion synthesis; K63, lysine 63

## Authors' contributions

The contents of this manuscript were written by JB and RKC and edited by BGW, WX and DAG. JB, RKC, BGW and DAG conceived and designed the experiments. Overall, JB, KH, RKC performed the experiments. JB, RKC, BGW, and DAG analyzed the data. JB and RKC contributed reagents/materials/analysis tools. All authors have read and approved the final manuscript.

## Supplementary Material

Additional File 1 **siRNA targeting of MMS2 and UEV1A**. **(A) **Hela cells and **(B) **293T cells were subjected to immunoblotting with an anti-Mms2/Uev1a antibody 72 hours post transfection of siRNAs targeting both MMS2 and UEV1A. Click here for file

Additional File 2 **siRNA targeting of UBC13, UEV1A and MMS2**. **(A) **Hela cells and **(B) **293T cells were subjected to immunoblotting with an anti-Ubc13 antibody 72 hours post transfection of siRNA targeting UBC13. **(C) **Hela cells and **(D) **293T cells were subjected to immunoblotting with an anti-Mms2/Uev1a antibody 72 hours post transfection of siRNAs targeting both MMS2 and UEV1A. Click here for file

Additional File 3 **PCNA ubiquitin laddering is not altered by the knockdown of UEV1a and MMS2**. Western blot analysis using an anti-PCNA antibody was performed on **(A) **Hela and **(B) **293T protein lysates from the same samples used in the immunoprecipitations for Figure 5. Click here for file
