# Supplementary material for: hMMS2 serves a redundant role in human PCNA polyubiquitination
Source: BMC Mol Biol. 2008 Feb 19;9:24. doi: 10.1186/1471-2199-9-24 (PMC2263069; doi:10.1186/1471-2199-9-24)
Supplement: Additional File 1 — siRNA targeting of MMS2 and UEV1A. (A) Hela cells and (B) 293T cells were subjected to immunoblotting with an anti-Mms2/Uev1a antibody 72 hours post transfection of siRNAs targeting both MMS2 and UEV1A. [file 1471-2199-9-24-S1.pdf]

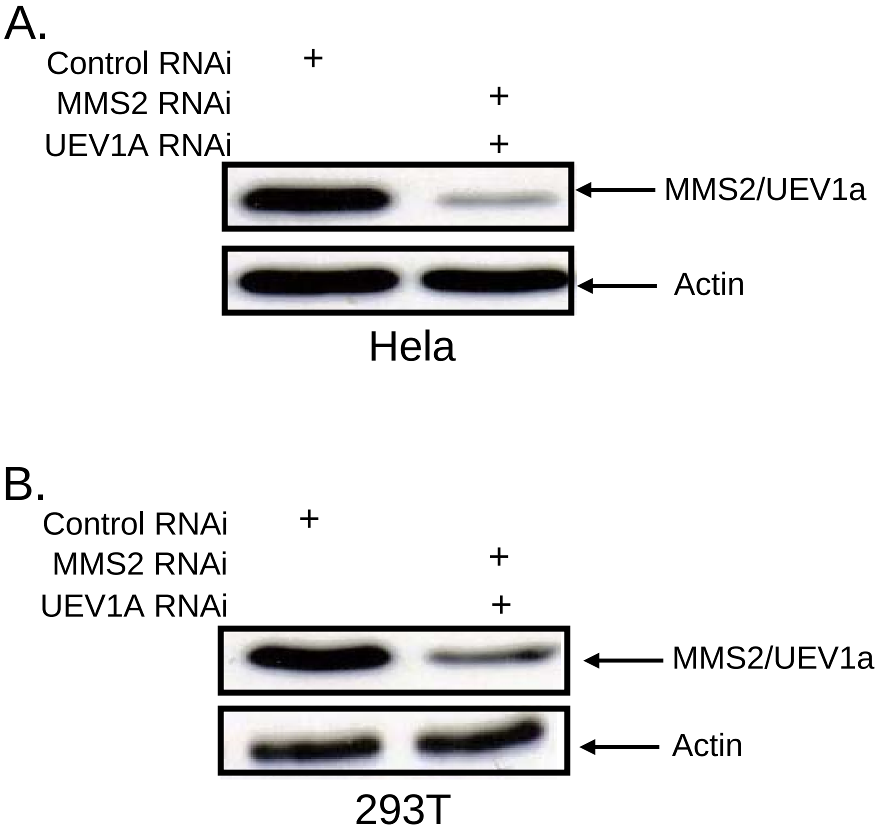
A.
Control RNAi
MMS2 RNAi
UEV1A RNAi
+
+
+
MMS2/UEV1a
Actin
Hela
B.
Control RNAi
MMS2 RNAi
UEV1A RNAi
+
+
+
MMS2/UEV1a
Actin
293T
