# Supplementary figures and images for: hMMS2 serves a redundant role in human PCNA polyubiquitination
Source: BMC Mol Biol. 2008 Feb 19;9:24. doi: 10.1186/1471-2199-9-24 (PMC2263069; doi:10.1186/1471-2199-9-24)

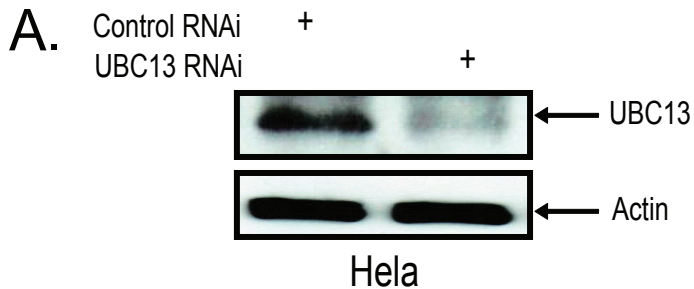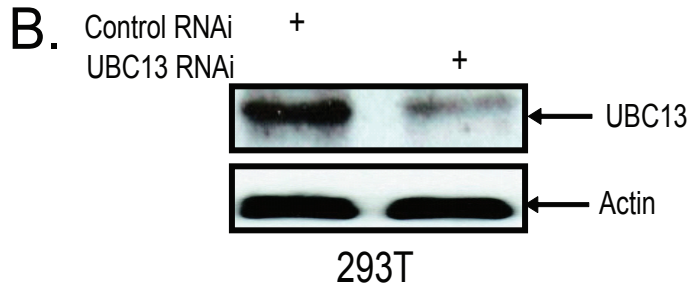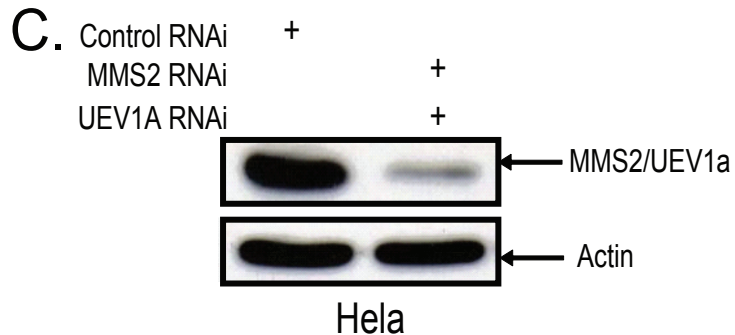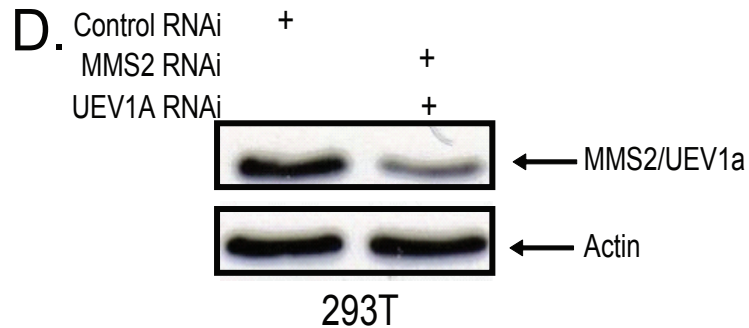

Supplement: Additional File 2 — siRNA targeting of UBC13, UEV1A and MMS2. (A) Hela cells and (B) 293T cells were subjected to immunoblotting with an anti-Ubc13 antibody 72 hours post transfection of siRNA targeting UBC13. (C) Hela cells and (D) 293T cells were subjected to immunoblotting with an anti-Mms2/Uev1a antibody 72 hours post transfection of siRNAs targeting both MMS2 and UEV1A. [file 1471-2199-9-24-S2.pdf]

A.

Hela

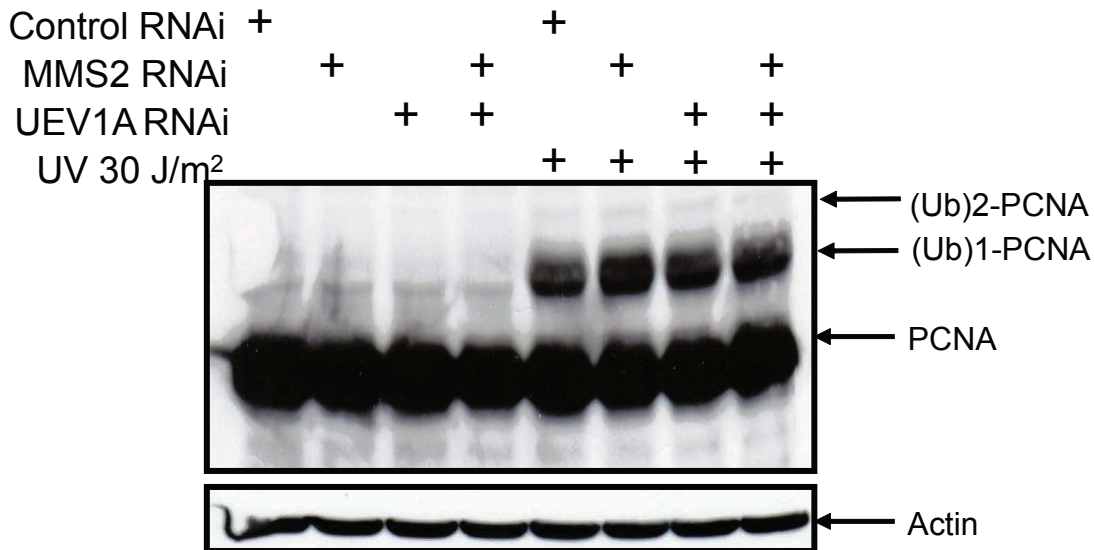

B.

293T

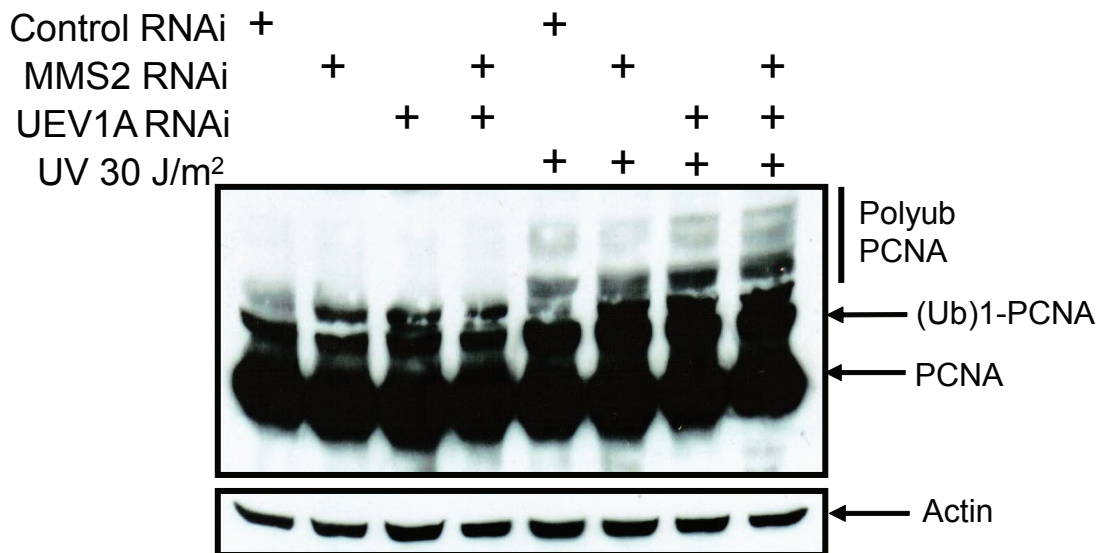

Supplement: Additional File 3 — PCNA ubiquitin laddering is not altered by the knockdown of UEV1a and MMS2. Western blot analysis using an anti-PCNA antibody was performed on (A) Hela and (B) 293T protein lysates from the same samples used in the immunoprecipitations for Figure 5. [file 1471-2199-9-24-S3.pdf]
